# Quality of Relationships between Informal Caregivers and Patients with Mild Cognitive Impairment

**DOI:** 10.1002/alz70858_096377

**Published:** 2025-12-24

**Authors:** Pascual Sanchez‐Juan, Elena Garcia Arcelay, Mariló Almagro, Mircea Balasa, Gerard Piñol‐Ripoll, Mercè Boada, Lamberto Landete, Inmaculada Abellán, Angel Berbel, Beatriz Espejo, Miguel Baquero, Juan Marin, Emilio Franco‐Macías, Alberto Villarejo Galende, Felix Viñuela Fernandez, Inmaculada Feria Vilar, Carmen Pérez‐Vieitez, Norberto Rodriguez‐Espinosa, Albert Puig‐Pijoan, Eduard Bargay Pizarro, Eloy Rodríguez Rodríguez, Jesús Rodrígo, Jorge Mauriño, María Sagrario Manzano

**Affiliations:** ^1^ Alzheimer's Centre Reina Sofia‐CIEN Foundation, Madrid, Spain; ^2^ Roche Farma S.A, Madrid, Madrid, Spain; ^3^ Confederación Española de Alzheimer y otras Demencias (CEAFA), Pamplona, Navarra, Spain; ^4^ Alzheimer's disease and other cognitive disorders unit, Hospital Clínic, IDIBAPS, Barcelona, Spain; ^5^ Cognitive Disorder Unit, Hospital Universitari Santa Maria, Lleida, Spain; ^6^ Research Center and Memory Clinic, ACE Alzheimer Center Barcelona, Universitat Internacional de Catalunya, Barcelona, Spain; ^7^ Department of Neurology, Hospital Universitario Dr. Peset, Valencia, Valencia, Spain; ^8^ Neurology and Dementia Unit, Hospital San Vicente del Raspeig, Alicante, Alicante, Spain; ^9^ Department of Neurology. Hospital de la Cruz Roja, Madrid, Madrid, Spain; ^10^ Cognitive‐Behavioral Neurology Unit Hospital Clínico San Cecilio, Granada, Granada, Spain; ^11^ Servei de Neurologia, Hospital Universitari i Politècnic La Fe, Valencia, Spain; ^12^ Dementia Unit, Hospital Clínico Universitario Virgen de la Arrixaca, Murcia, Murcia, Spain; ^13^ Unidad de Demencias, Servicio de Neurología y Neurofisiología. Instituto de Biomedicina de Sevilla (IBiS), Hospital Universitario Virgen del Rocío/CSIC/Universidad de Sevilla, Sevilla, Spain; ^14^ Hospital Universitario 12 de Octubre Research Institute, Madrid, Spain; ^15^ Instituto Neurológico Andaluz. Hospital Victoria Eugenia/Unidad Deterioro Cognitivo. Hospital Virgen Macarena, Sevilla, Spain; ^16^ Department of Neurology, Complejo Hospitalario Universitario de Albacete, Castilla‐La Mancha, Albacete, Albacete, Spain; ^17^ Department of Neurology, Hospital Universitario de Gran Canaria Doctor Negrín, Las Palmas de Gran Canaria, Las Palmas de Gran Canaria, Gran Canaria, Spain; ^18^ Department of Neurology, Hospital Universitario Nuestra Señora de Candelaria Santa Cruz de Tenerife, Santa Cruz de Tenerife, Tenerife, Spain; ^19^ Servei de Neurologia, Hospital del Mar, Barcelona, Spain; ^20^ Son Espases University Hospital, Palma, Spain; ^21^ Hospital Universitario Marqués de Valdecilla – IDIVAL – University of Cantabria, Santander, Cantabria, Spain; ^22^ Neurology Department Infanta Leonor Hospital, Madrid, MADRID, Spain

## Abstract

**Background:**

The quality of the caregiver‐patient relationship is essential for both parties, as it significantly influences the emotional well‐being of patients, and the satisfaction and burden of caregivers. This study aimed to assess the quality of caregiver‐patient relationships in informal caregivers of patients with mild cognitive impairment (MCI) and to identify associated factors.

**Method:**

A non‐interventional, cross‐sectional study was conducted at 19 memory clinics in collaboration with the Spanish Confederation of Alzheimer's Disease (CEAFA). Informal caregivers of patients with MCI (NIA‐AA criteria) and a Global Deterioration Scale score of 3 were included. The quality of the patient‐caregiver relationship was measured using the Quality of Carer–Patient Relationship Scale (QCPR). A score >56 indicates a good‐quality relationship. Associations between QCPR scores and caregiver demographic, psychological, and behavioral factors, as well as patient characteristics, were analyzed through logistic regression.

**Result:**

A total of 196 caregivers were studied (mean age: 63.5 ± 13.1 years; 63% female). Of these, 68.4% were spouses of patients (mean age: 72.9 ± 7.0 years) with a median disease duration of 2 years (IQR 1.0‐4.0) and a median MMSE score of 26.0 (IQR 23.0‐27.0).

Over half of the participants reported a good relationship with the patient (53.6%, mean QCPR score: 26.1 ± 6.1). Higher QCPR scores were associated with lower caregiving burden, greater care competence, better quality of life, stronger social support, greater resilience, and higher levels of anxiety and depression. Younger caregivers, employed, and living separately from the patient reported having better relationships with them.

In multivariate analysis, a good patient‐caregiver relationship was independently associated with higher MMSE score (OR=1.165, 95% CI 1.034‐1.312, *p* = 0.012), fewer neuropsychiatric symptoms (OR=0.865, 95% CI 0.795‐0.942, *p* = 0.001), lower use of avoidant coping strategies (OR=0.424, 95% CI 0.182‐0.987, *p* = 0.047), and lower perception of stigma (OR=0.048, 95% CI 0.048‐0.007, *p* = 0.002).

**Conclusion:**

The quality of the relationship is influenced by demographic, psychological, and behavioral factors relating to both the caregiver and the patient. Specific interventions on these factors could be useful in enhancing caregiver‐patient relationships.

